# Combined application of manure and straw with chemical fertilizers enhances soil quality and maize yield by regulating bacterial community composition and diversity: a long-term field study

**DOI:** 10.3389/fpls.2026.1881326

**Published:** 2026-07-23

**Authors:** Wenbo Mi, Shuying Wang, Jianjun Zhang, Gang Zhao, Yi Dang, Lei Wang, Gang Zhou, Xujiao Zhou, Jingyu Hu, Tinglu Fan, Shangzhong Li

**Affiliations:** 1 Institute of Dryland, Gansu Academy of Agricultural Sciences, Lanzhou, China; 2 Key Laboratory of Low Carbon Green Agriculture in Northwestern China, Ministry of Agriculture and Rural Affairs, Lanzhou, China; 3 Key Laboratory of Higher Water Utilization on Dryland of Gansu Province, Lanzhou, China

**Keywords:** bacterial diversity, organic material addition, soil properties, soil quality index, yield

## Abstract

**Introduction:**

Against the backdrop of excessive chemical fertilizer application and challenges to global food security, promoting the development of sustainable green agriculture has become urgently necessary.

**Methods:**

Based on a long-term field experiment, this study investigated the relationships between microbial communities and soil quality under different fertilization practices. Six fertilization treatments were established: no fertilizer (CK), mineral nitrogen alone (N), combined mineral nitrogen and phosphorus (NP), manure alone (M), combined mineral fertilizer and manure (MNP), and combined mineral fertilizer and straw (SNP).

**Results:**

MNP and SNP significantly increased the soil quality index (SQI) and maize yield, with MNP showing greater improvement than SNP. Soil organic carbon (SOC), total nitrogen (TN), and microbial biomass carbon (MBC) were identified as the main contributors to SQI and maize yield. The relative abundances of dominant bacterial phyla and genera increased under MNP and SNP treatments, among which *Pseudomonadota* and *Lysobacter* showed significant correlations with soil properties. Furthermore, the MNP treatment led to an increase in bacterial alpha diversity. Regression analysis showed that bacterial alpha diversity was positively correlated with SOC, TN, Olsen-P, MBN, MBC, and mineral nitrogen content, while negatively correlated with soil pH. Both bacterial community composition and diversity positively influenced SQI. The main factors driving the increase in bacterial diversity were soil pH, NO_3_^-^-N, NH_4_^+^-N, and Olsen-P.

**Discussion:**

In conclusion, MNP and SNP enhanced soil quality and crop yield by optimizing soil properties, thereby increasing bacterial diversity and the abundance of dominant bacterial communities. The study provides a deeper theoretical basis for the optimization of organic fertilization techniques and the regulation of soil health.

## 1 Introduction

The rapid development of intensive agriculture has led to severe soil degradation in farmland, posing a threat to global food security (Islam et al., 2022; Raven and Wagner, 2021). Extensive research indicates that the widespread use of chemical fertilizers restricts microbial community composition and diversity while reducing crop productivity (Hu et al., 2026; Sun et al., 2025). Microorganisms, including fungi and bacteria, are considered key drivers of nutrient cycling and play a vital role in accelerating soil organic matter transformation and increasing nutrient mineralization rates (Luo et al., 2023). These factors directly or indirectly influence soil quality and crop yields (Xuan et al., 2009). Therefore, regulating the structure of soil microbial communities in agricultural ecosystems through improved fertilization practices is crucial for enhancing soil quality and crop productivity.

The combined application of organic materials (e.g., manure and straw) with chemical fertilizers is an important practice in sustainable agriculture, as it alleviates long-term challenges such as soil degradation, decline in organic matter, and loss of microbial diversity caused by the long-term application of chemical fertilizers alone (Lv et al., 2024; Meng et al., 2021). The integration of organic materials with chemical fertilizers is widely advocated to synergistically improve soil fertility and crop productivity (Lyu et al., 2025b). Soil microorganisms govern the biogeochemical cycles of carbon, nitrogen, and phosphorus, processes that are closely associated with organic matter decomposition and nutrient release (Wang et al., 2024). The combined application of manure or straw with chemical fertilizers significantly promotes the proliferation and activity of bacterial and fungal communities in agricultural soils (Gao et al., 2021; Tian et al., 2022). Furthermore, studies have shown that the combined application of organic materials and chemical fertilizers increases soil aggregate stability and enhances microbial biomass carbon and nitrogen in both bulk and rhizosphere soils (Hontoria et al., 2019). Recently, it has been reported that long-term combined application of manure or straw with chemical fertilizers strengthens the symbiotic relationship between arbuscular mycorrhizal fungi and crop roots, promoting the release of organic acids and phosphatases in root exudates, thereby improving the uptake efficiency of nitrogen and phosphorus. In addition, studies have confirmed that the combined application of organic materials and chemical fertilizers significantly increases soil organic carbon stocks and enzyme activities (Yu et al., 2026; Zhang et al., 2026). However, systematic studies on the structural characteristics and ecological functions of bacterial and fungal communities, as well as their regulatory mechanisms on soil quality under long-term combined application of organic materials with chemical fertilizers, remain limited. In particular, the differential effects of different organic material types (manure vs. straw) combined with chemical fertilizers are still unclear.

Soil microbial community composition and diversity are regulated by multiple factors, including soil physicochemical properties (Linton et al., 2020). As important supplementary sources of soil organic matter, manure and straw influence microbial community structure and function by supplying organic matter and nutrients to the soil. Meanwhile, the addition of manure and straw improves soil aggregate structure, reduces bulk density, and significantly increases soil total nutrient content. These improvements create favorable habitats for microbial proliferation (D’Amours et al., 2023; Yang et al., 2019). Furthermore, pH is a crucial environmental factor highly sensitive to microbial activity. The addition of organic materials buffers soil pH, thereby making the microbial community more diverse and stable (Giannetta et al., 2018). Notably, compared with chemical fertilizer alone, the combined application of manure and straw with chemical fertilizers exhibits synergistic effects in increasing soil organic carbon stocks and optimizing microbial community structure. Among these, manure, due to its higher content of stable organic matter, is more conducive to long-term soil organic carbon sequestration, whereas straw, with its higher carbon-to-nitrogen ratio, may induce microbial nitrogen competition during the initial decomposition stage, although long-term combined application with chemical fertilizers can alleviate this issue (Chen et al., 2023; Liu et al., 2025; Zhang et al., 2026). In summary, the combined application of manure and straw with chemical fertilizers can directly affect soil microbial communities by serving as available carbon and nutrient sources, and indirectly by modifying soil physicochemical properties and creating a more favorable soil environment for microbial growth and interactions, thereby comprehensively improving soil quality and crop productivity.

It is well known that microorganisms acquire the energy required for their activities through the decomposition and consumption of carbon derived from plant residues (Zhao et al., 2026a). The addition of manure and straw can accelerate microbial metabolism and is decomposed by microorganisms after being incorporated into the soil (Ma et al., 2024; Zhao et al., 2026a). Based on this, we hypothesize that long-term addition of manure and straw can improve soil quality and crop productivity by increasing the abundance and diversity of soil-dominant microbial communities. However, studies investigating the effects of manure and straw addition on soil microbial communities have largely focused on specific utilization patterns (Ma et al., 2025; Sun et al., 2025). These studies have not extensively assessed long-term soil quality through comprehensive evaluations considering multiple physical, chemical, and biological factors. Furthermore, the mechanisms governing the effects of manure and straw addition on soil microbial community composition and diversity, and their subsequent influence on soil quality, are not yet fully understood. In particular, the differential effects of different organic material types and their synergistic mechanisms with chemical fertilizers require further investigation. This study aimed to: 1) quantitatively evaluate soil quality under different organic material management strategies by assessing soil physicochemical properties using the total dataset method; 2) investigate the correlations between soil bacterial and fungal communities, characteristics of dominant microbial taxa, and soil properties following long-term addition of manure and straw; 3) explore the effects of microbial community composition and diversity on soil quality, and elucidate the underlying mechanisms by which manure and straw addition regulate soil health through microbial mediation, thereby providing a theoretical basis for optimizing organic fertilization practices.

## 2 Materials and methods

### 2.1 Experimental site

This study was based on a long-term fertilizer field experiment conducted in Gaoping, Pingliang, Gansu Province, located on the Loess Plateau of Northwest China (35°16′ N, 107°30′ E; elevation 1,150 m). The experimental site has an average annual temperature of 8.0 °C, annual evaporation of 1,380 mm, and average annual precipitation of 540 mm, of which 60% falls during the three months of July, August, and September. The test soil was a dark loessial soil. At the beginning of the long-term experiment (autumn 1978), the soil had an organic carbon content of 5.24 g·kg^-^¹, total nitrogen of 1.00 g·kg^-^¹, total phosphorus of 0.57 g·kg^-^¹, alkali-hydrolyzed nitrogen of 65.9 mg·kg^-^¹, available phosphorus of 6.77 mg·kg^-^¹, available potassium of 163 mg·kg^-^¹, and a pH of 8.20. The soil particle size composition consisted of 23.14% sand (2–0.02 mm), 43.21% fine sand (0.02–0.002 mm), and 33.65% clay (<0.002 mm).

### 2.2 Experimental design and management

The experiment included six treatments: (1) no fertilizer (CK); (2) nitrogen fertilizer alone at 90 kg·ha^-1^ (N); (3) nitrogen fertilizer at 90 kg·ha^-1^ plus phosphorus fertilizer at 75 kg·ha^-1^ (NP); (4) farmyard manure alone at 75 t·ha^-1^ (M); (5) straw incorporation at 3.75 t·ha^-1^ combined with nitrogen at 90 kg·ha^-1^ and phosphorus at 75 kg·ha^-1^ (SNP); (6) farmyard manure at 75 t·ha^-1^ combined with nitrogen at 90 kg·ha^-1^ and phosphorus at 75 kg·ha^-1^ (MNP). The nutrient contents of the farmyard manure were: N 1.6 g·kg^-^¹, P_2_O_5_ 1.6 g·kg^-^¹, and K_2_O 14.8 g·kg^-^¹. In the SNP treatment, phosphorus fertilizer was applied once every two years. After each crop harvest, the straw was cut into approximately 5 cm segments and incorporated into the soil through deep plowing. The straw incorporation rate of 3.75 t·ha^-1^ was equivalent to an input of 1,600 kg C·ha^-1^. In the other treatments, phosphorus fertilizer, organic manure, and 60% of the nitrogen fertilizer were applied as basal fertilizer before land preparation, while the remaining 40% of nitrogen fertilizer was applied as topdressing. The experiment followed a one-crop-per-year rotation system: spring maize (continuous cropping for two years) → wheat (continuous cropping for four years). Each treatment occupied an area of 666.7 m², with no replication, and the plots were arranged sequentially in a large-plot design. Spring maize was sown by hole-sowing without film mulching at a density of 52,500 plants·ha^-1^, while wheat was sown by mechanical drilling at a density of 187.50 kg·ha^-1^. This study mainly utilized data from the spring maize growing seasons of 2024 and 2025.

### 2.3 Sampling and measurement

#### 2.3.1 Collection and analysis of soil samples

In September 2024 and 2025, during the maize harvest period, soil samples were collected from the 0–30 cm layer (stratified at 10 cm intervals). After combining the soil samples from each layer and passing them through a 2 mm mesh, each composite sample was split into three portions. The first subsample was air-dried at room temperature for the determination of soil organic carbon (SOC), total nitrogen (TN), Olsen−phosphorus (Olsen−P), and pH. The second subsample was stored at 4 °C for the determination of ammonium−N (NH_4_^+^-N) and nitrate−N (NO_3_^-^-N). The third subsample was stored at −20 °C for the determination of microbial biomass carbon (MBC), microbial biomass nitrogen (MBN), and microbial sequencing. SOC was measured using the K_2_Cr_2_O_7_–H_2_SO_4_ oxidation method (Walkley, 1935). TN was analyzed using an elemental analyzer. The pH was determined using a pH meter with a soil-to-water ratio of 1:2.5. NH_4_^+^-N and NO_3_^-^-N contents were measured using a flow injection analyzer. MBN and MBC were quantified using the chloroform fumigation−extraction method (Vance et al., 1987). In addition, three points within each plot were selected to excavate soil profiles (0–30 cm), and soil bulk density (BD) was determined using the core method.

#### 2.3.2 Assessment of soil quality

For the measured indicators, the total data set (TDS) method was used to evaluate the soil quality index (SQI) following the approach described by Doran and Parkin (1994). Principal component analysis (PCA) was employed to determine the proportion of variance explained by each factor within the common factor variance, and these proportions were then used as weights for each indicator (Shukla et al., 2006). A nonlinear scoring function was applied to standardize and assign scores to the soil physicochemical indicators, as follows:


$$FNL=\frac{x}{1+{\left(x/x_{i}\right)}^{m}}$$

where FNL represents the nonlinear transformation score of each indicator; x represents the actual value of the indicator; and x_i_ represents the mean value of the indicator. The scores of each indicator were determined in two opposite directions, with m = -2.5 denoting a “more is better” function and m = 2.5 denoting a “less is better” function. The soil quality index (SQI) can be calculated as follows (Andrews et al., 2002):


$$SQI=\sum_{i=1}^{n}W_{i}\times FNL_{i}$$

where *FNL_i_* represents the score of indicator *i*; *n* represents the number of indicators, and Wi represents the weight of indicator *i*.

#### 2.3.3 Maize yield

The spring maize was harvested individually from each plot when the maize crop reached maturity. Then, it was air-dried, threshed, and weighed. The grain exhibited a moisture content of 14% at the time of threshing.

#### 2.3.4 Sequencing of bacterial and fungal communities in the soil

After the soil sample DNA was extracted, the V3+V4 region of the 16S rDNA was amplified using specific primers with barcodes. The primer sequences were: 341F: CCTACGGGNGGCWGCAG; 806R: GGACTACHVGGGTATCTAAT. Amplify the ITS2 region of the ITS using barcoded, species-specific primers. The primer sequences for ITS were: ITS3_KYO2: GATGAAGAACGYAGYRAA; ITS4: TCCTCCGCTTATTGATATGC. The purified amplification products were ligated with sequencing adapters to construct a sequencing library, and then subjected to on−board sequencing using the Illumina platform.

After obtaining the raw reads from sequencing, we first used DADA2 to filter and correct the reads, and output non−redundant reads with their corresponding abundance information. The reads were then merged into tags, followed by the removal of chimeric tags to obtain the tag sequences and abundances for subsequent analysis, i.e., ASV sequences and ASV abundance information. Based on the ASV sequences and abundance data, we performed species annotation, species composition analysis, indicator species analysis, alpha diversity analysis, beta diversity analysis, and community function prediction (Chao, 1984; Shannon, 1948; Simpson, 1949). Microbial sequencing was completed by Guangzhou Aozhi Biotechnology Co., Ltd., and the raw data of the sequenced microorganisms have been deposited in the NCBI SRA under accession number PRJNA1465624.

### 2.4 Statistical analysis of data

The values presented in this study are the means obtained from the three soil layers. The data were tested for normality and homogeneity of variance. Considering that the field treatments were arranged as large plots (each 666.7 m²) without field replication, the statistical significance of treatment effects was evaluated using one−way analysis of variance (ANOVA) based on intra−plot sampling replicates. Specifically, for each treatment area, three independent sub−samples collected from fixed sampling points were treated as the analytical units (n = 3). The ANOVA model was constructed with ‘Treatment’ as the fixed factor and the variance among the sub−samples within each plot as the random error term. This approach allows for the detection of treatment differences under the assumption that the within−plot heterogeneity is representative of the random experimental error. To account for potential spatial trends along the sequential layout, we performed a linear regression analysis on the pre−treatment soil properties against the plot sequence numbers; no significant gradient was detected (*P* > 0.05), justifying the sequential design. Following ANOVA, multiple comparisons among treatments were conducted using the least significant difference (LSD) test at the *P* < 0.05 significance level. Mantel tests were conducted in RStudio using the corrplot, tidyverse, and ggplot2 packages, while structural equation modeling (SEM) was carried out with the piecewiseSEM package. All figures were generated using Origin 2021 and R 4.4.2 (https://cran.r-project.org).

## 3 Results

### 3.1 Soil properties

Manure fertilizer and straw addition (MNP and SNP) significantly elevated the levels of TN, Olsen-P, NH_4_^+^-N, NO_3_^-^-N, SOC, MBN, and MBC, while decreasing pH and BD in comparison to the regular fertilization (NP). The Olsen-P, NO_3_^-^-N, SOC, and MBN levels were higher under MNP than under SNP, with significant increases of 59.41%, 71.18%, and 10.90%, respectively. It is noteworthy that the SOC content under the MNP treatment was significantly higher than that under the M treatment. This is primarily attributed to the fact that the MNP treatment incorporates both the direct carbon input from organic manure and the indirect effects resulting from chemical fertilizers promoting crop growth and increasing root biomass, thereby creating a positive feedback loop that accelerates soil organic carbon accumulation. In long-term field experiments, this cumulative effect will be further amplified, making the differences between treatments even more pronounced.

### 3.2 Soil quality and maize yield

**Figure 1:**
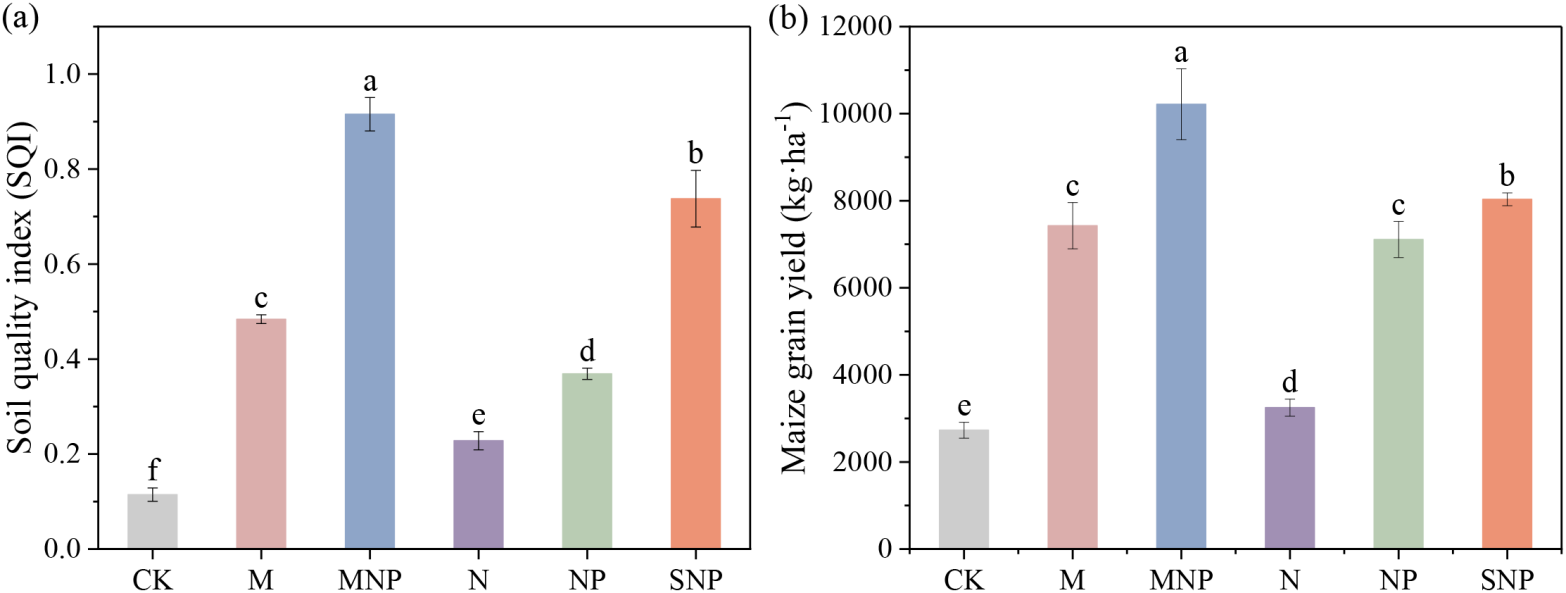
Soil quality index and maize grain yield under different fertilizer management strategies. **(a)** Soil quality index. **(b)** Maize grain yield. Significant differences among treatments are indicated in different lowercase letters (*P<* 0.05).

The complete dataset included 10 soil parameters for evaluating the soil quality index (SQI). The rankings of SQI and maize yield under different fertilization management practices were as follows: MNP > SNP > M > NP > N > CK (**Figures 1a, b**). Compared with NP, the SQI values under MNP and SNP treatments were 145.95% and 99.95% higher, respectively. Furthermore, the maize yield under MNP and SNP treatments was 43.68% and 12.99% higher, respectively, compared with NP.

**Figure 2:**
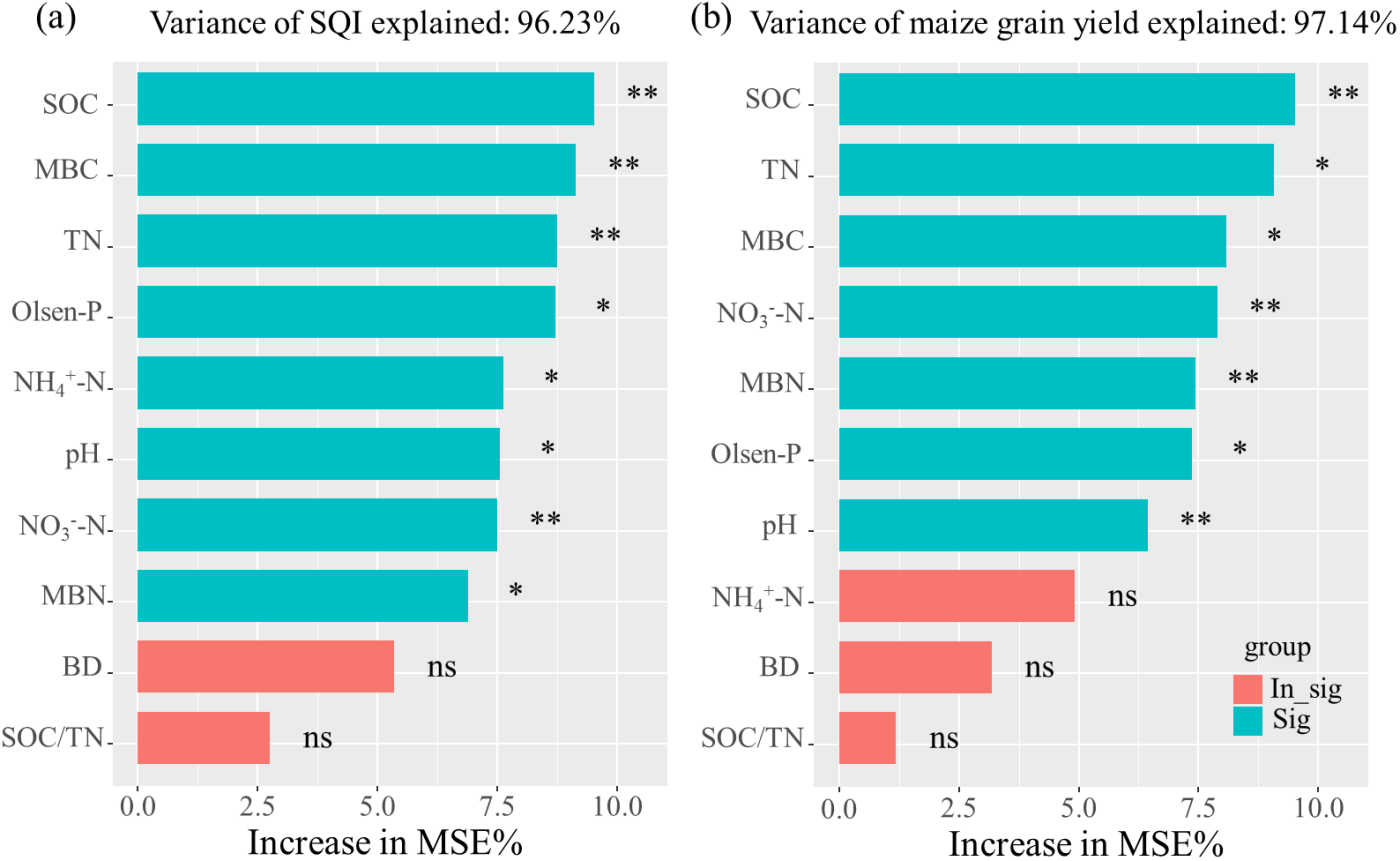
Contribution of soil factors to SQI **(a)** and maize grain yield **(b)**. **P < 0.01; *P < 0.05; and ns *P* > 0.05.

Random forest analysis revealed that the soil properties represented by SOC, MBC, TN, and Olsen-P made the most significant contributions to the SQI (**Figure 2a**). The soil properties represented by SOC, TN, MBC, and NO_3_^-^-N made the most significant contributions to maize yield (**Figure 2b**).

### 3.3 Characteristics of the soil microbial community

#### 3.3.1 Composition and diversity of species

**Figure 3:**
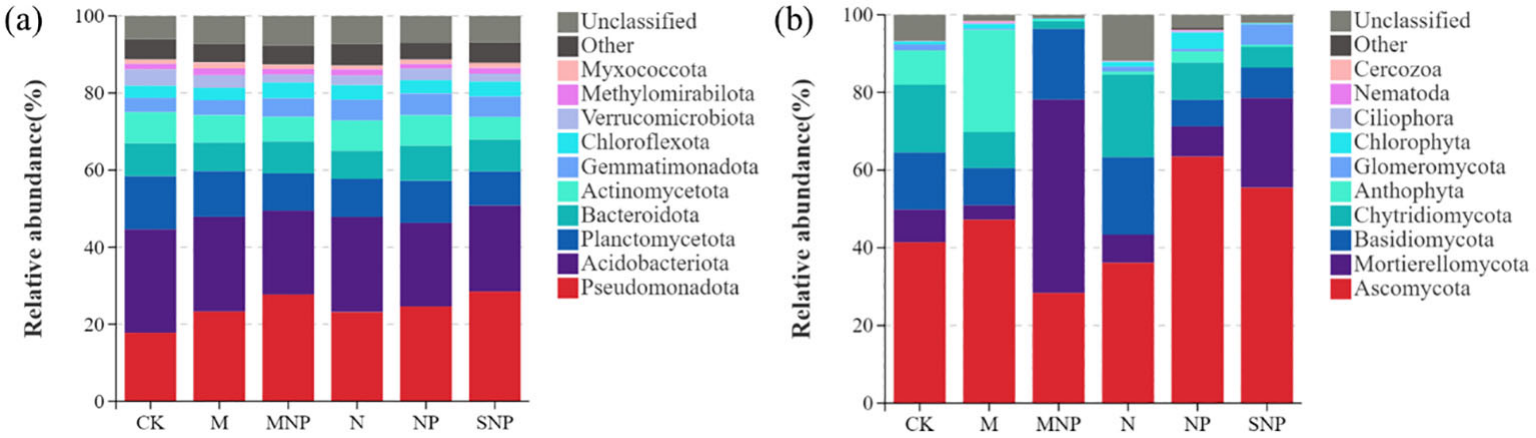
Composition of soil bacterial and fungal communities at phylum level under different fertilizer management strategies. **(a)** soil bacterial composition. **(b)** soil fungal composition.

In the soil bacterial community, *Pseudomonadota*, *Acidobacteriota*, *Planctomycetota*, *Bacteroidota*, and *Actinomycetota* were identified as the dominant phyla (**Figure 3a**). Compared with NP, the relative abundances of *Pseudomonadota* under the MNP and SNP treatments increased by 12.36% and 15.44%, respectively. The relative abundance of *Acidobacteriota* under the CK treatment was higher than that under the MNP and SNP treatments, by 23.22% and 20.23%, respectively. At the genus level, similar patterns were observed in the bacterial communities across treatments. *Sphingomonas* and *Lysobacter* were dominant in the MNP and SNP treatments (**Supplementary Figure 1a**, **Supplementary Data**).

The predominant fungal phyla in the soil included *Ascomycota*, *Mortierellomycota*, *Basidiomycota*, *Chytridiomycota*, and *Anthophyta*, collectively accounting for over 80% of the total fungal community (**Figure 3b**). Compared with CK and NP, the MNP treatment resulted in a decreased relative abundance of *Ascomycota*, while the relative abundances of *Mortierellomycota* and *Basidiomycota* increased. At the genus level, *Mortierella* exhibited a higher abundance under the MNP and SNP treatments than under the other treatments. Notably, *Pterula* showed high abundance only under the MNP treatment (**Supplementary Figure 1b**, **Supplementary Data**).

**Figure 4:**
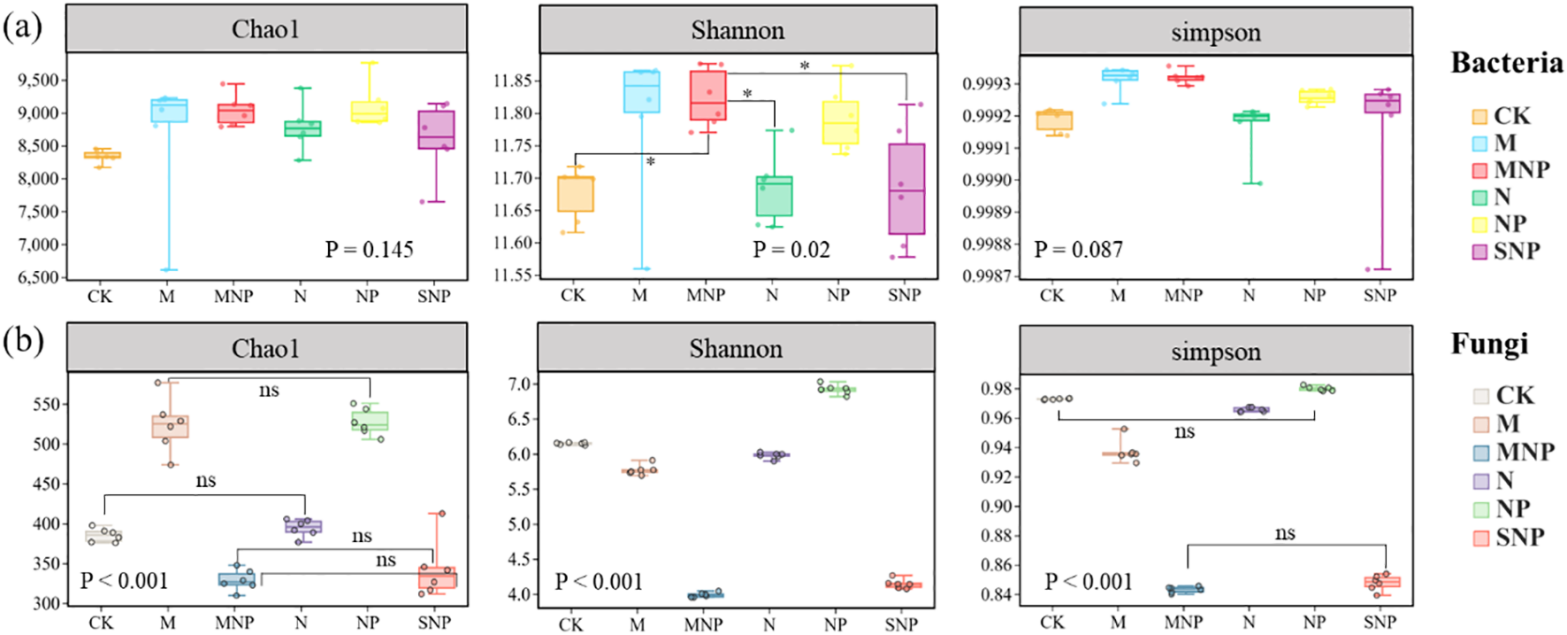
Alpha diversity of soil bacterial and fungal communities under different fertilizer management strategies. **(a)** soil bacterial diversity. **(b)** soil fungal diversity. Chao1, Shannon, and Simpson index are used to represent the alpha diversity of microbial communities. *P < 0.05; ns, *P* > 0.05.

Compared with CK and NP, the MNP treatment increased soil bacterial diversity (**Figure 4a**). Furthermore, the MNP and SNP treatments significantly reduced soil fungal diversity (*p* < 0.05), while no statistically significant difference in fungal community diversity was observed between the MNP and SNP treatments (*p* > 0.05) (**Figure 4b**).

#### 3.3.2 Differences among species

**Figure 5:**
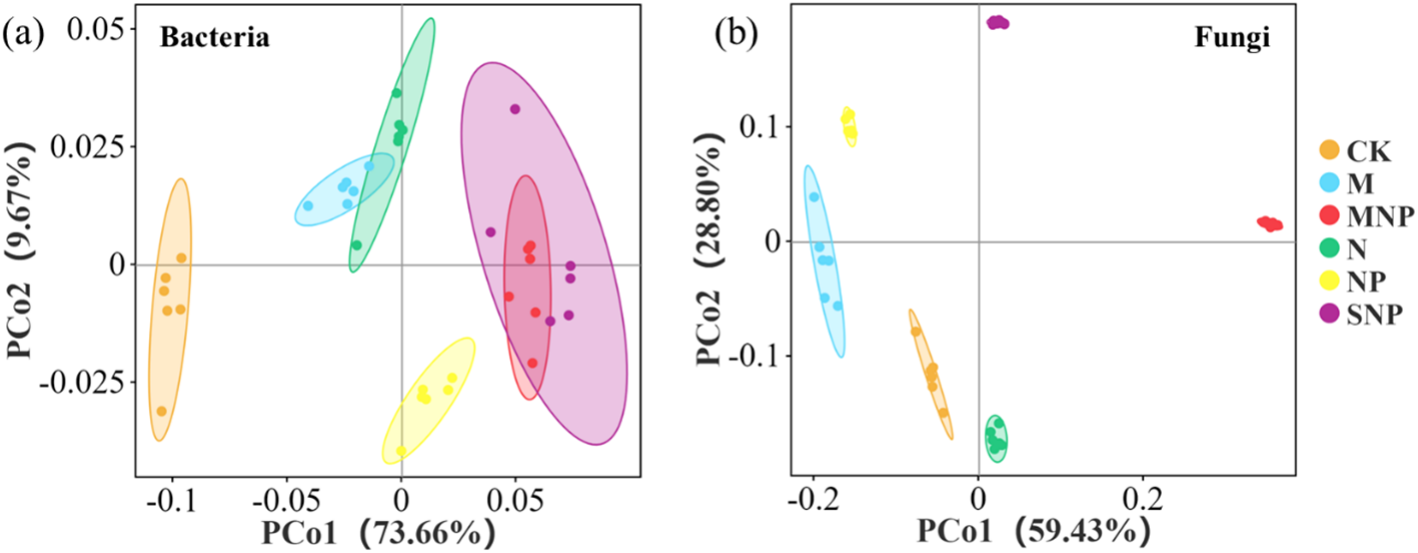
PCoA and of soil bacterial and fungal communities under the different fertilizer management strategies. **(a)** PCoA of soil bacteria. **(b)** PCoA of soil fungi.

According to the PCoA analysis, significant changes in microbial community composition were observed across different treatments. PCo1 and PCo2 explained 73.66% and 9.67% of the bacterial community variation, respectively (**Figure 5a**). Clear differences were observed among treatments. The MNP and SNP treatments exhibited highly similar bacterial communities, while the N and M treatments also showed highly similar bacterial communities. However, for the fungal community, the results revealed low similarity among different treatments (**Figure 5b**). Similar results were also obtained from the PCA analysis (**Supplementary Figure 2**, **Supplementary Data**).

#### 3.3.3 Relationships between soil properties and microbial characteristics

**Figure 6:**
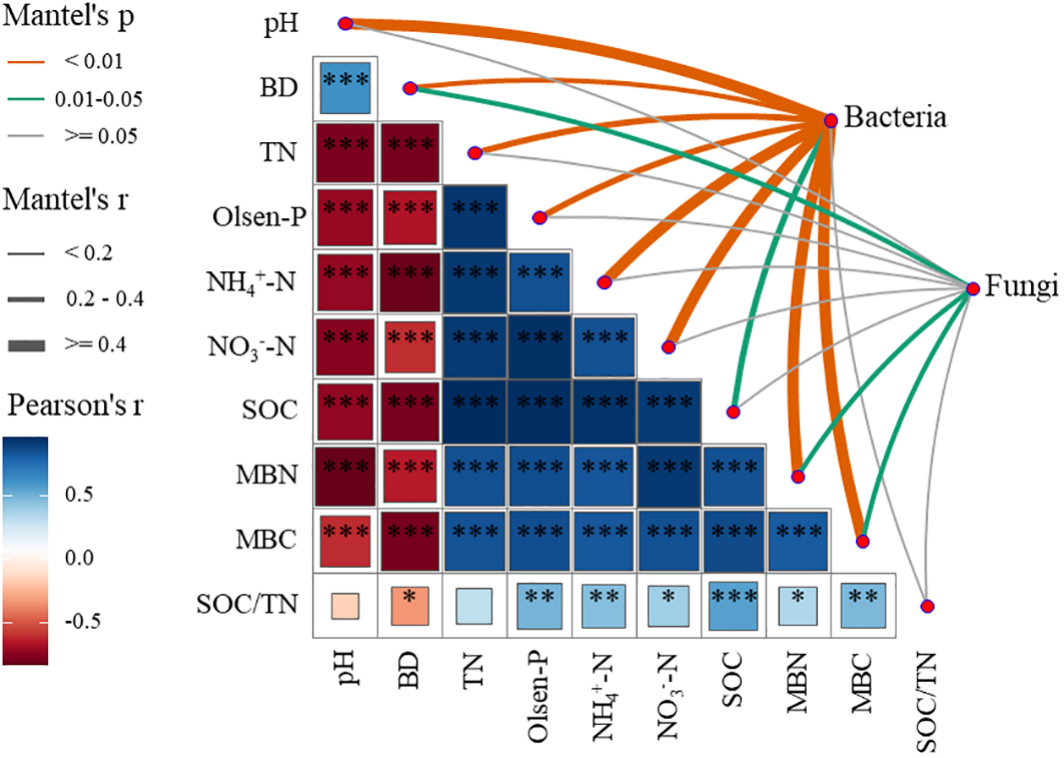
Mantel test showing soil factor correlations with soil bacterial and fungal communities. The width and thinness of the lines indicate the strength of the relationship between soil microbial community and soil factors. The color of the squares indicates the correlation between soil factors. *P < 0.05; **P < 0.01; ***P < 0.001.

Mantel tests revealed that environmental factors significantly influenced bacterial community composition (*p* < 0.05). However, the correlations between fungal community composition and environmental factors were relatively weak (**Figure 6**). Meanwhile, strong associations were observed among the environmental factors. For example, soil pH exhibited an extremely significant (*p* < 0.01) positive correlation with soil BD (*p* < 0.05), while both pH and BD showed negative correlations with all other environmental factors. With the exception of pH and BD, all environmental factors exhibited significant (*p* < 0.05) or extremely significant (*p* < 0.01) positive correlations with one another.

At the genus level, RDA1 and RDA2 explained 83.34% and 9.59% of the variation in bacterial community composition, respectively (**Supplementary Figure 3a**, **Supplementary Data**). The dominant bacteria in the MNP and SNP treatments were negatively correlated with pH and BD, while positively correlated with the other factors. *Sphingomonas* and *Lysobacter*, belonging to *Pseudomonadota*, exhibited the most significant correlations with environmental factors and were also closely associated with biological characteristics such as MBC and MBN (*p* < 0.05).

**Figure 7:**
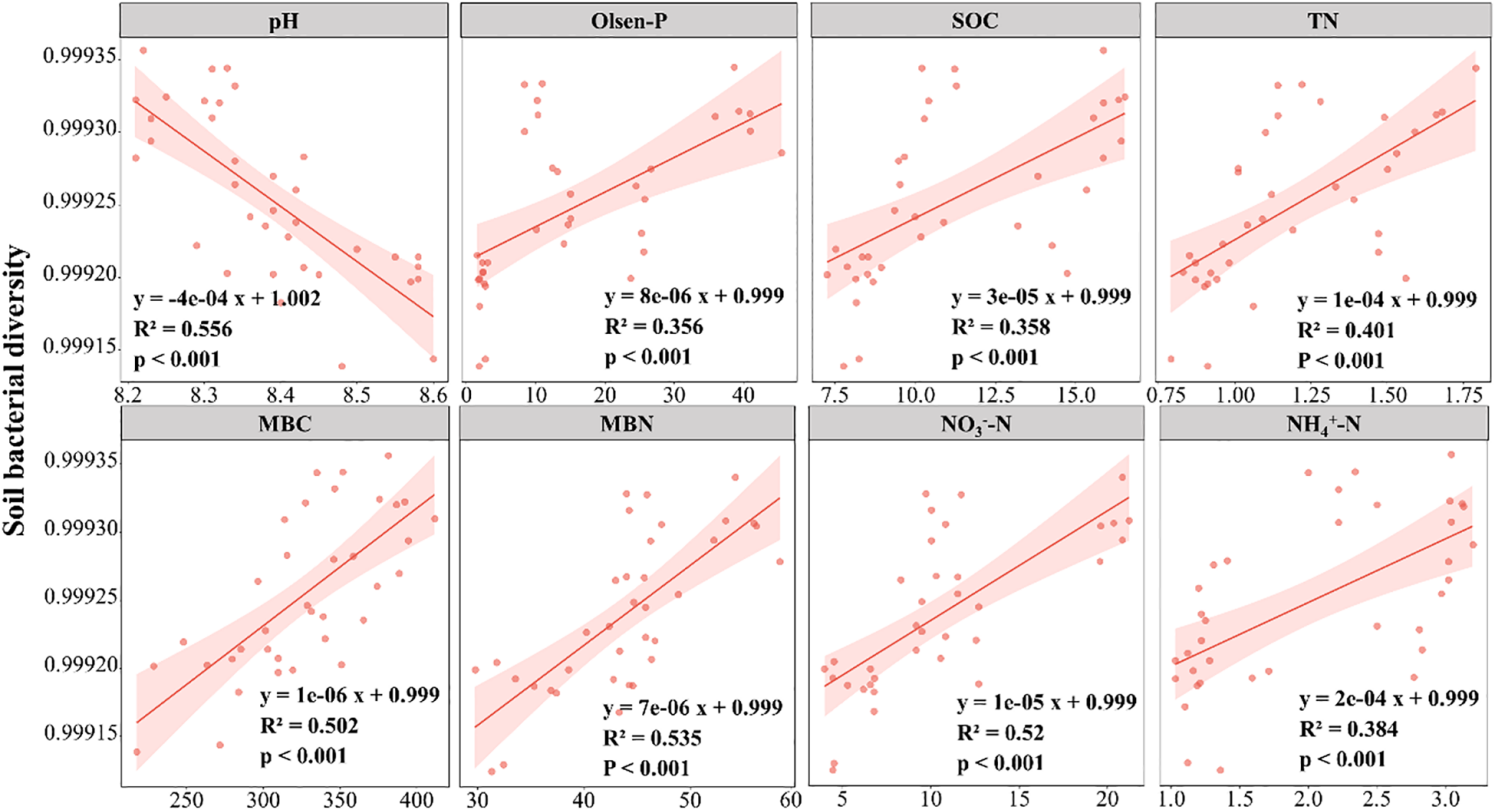
Correlation between soil bacterial communities and soil factors.

RDA1 and RDA2 explained 45.84% and 7.35% of the variation in fungal community composition, respectively (**Supplementary Figure 3b**, **Supplementary Data**). Therefore, manure addition primarily influenced the soil bacterial community by altering soil environmental factors. Regression analysis indicated that soil bacterial diversity was positively correlated with Olsen-P, SOC, TN, MBN, NO_3_^-^-N, NH_4_^+^-N, and MBC, while negatively correlated with soil pH (**Figure 7**).

### 3.4 Comprehensive effects of soil factors and bacterial community characteristics on SQI

**Figure 8:**
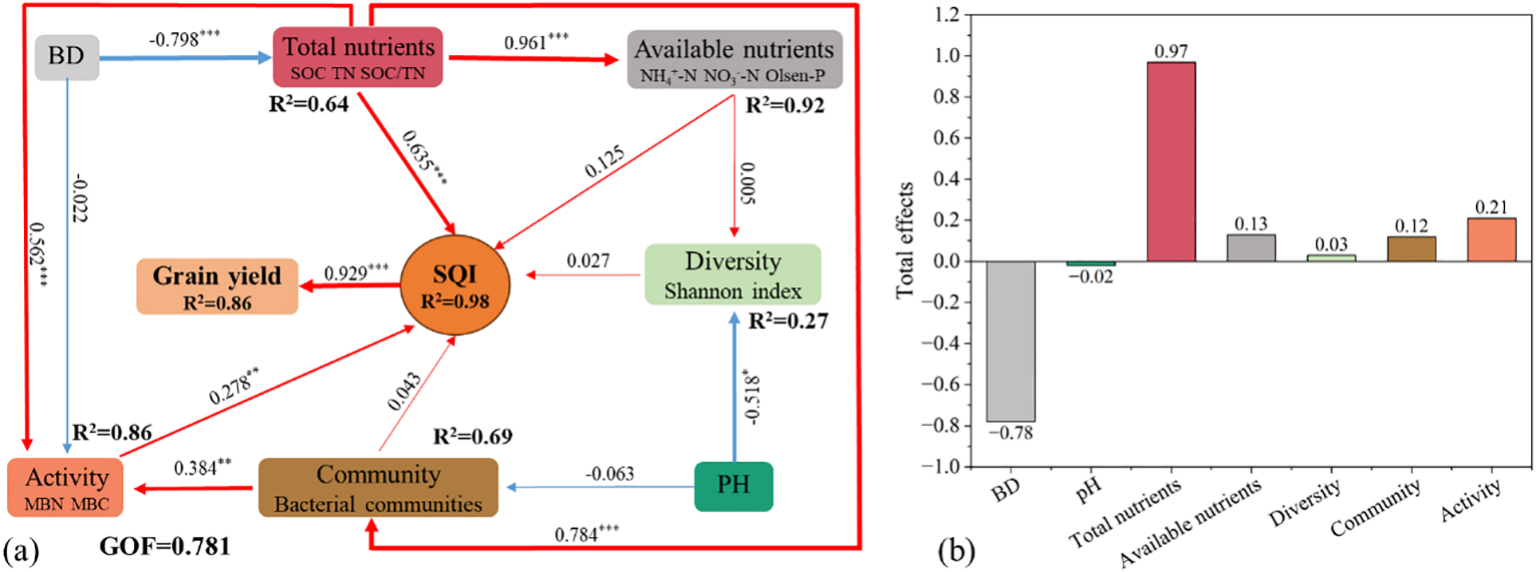
Pathways showing how the different soil properties impact the soil quality index **(a)**. Blue and red arrows represent significant negative and positive relationships, respectively (*P < 0.05, **P < 0.01, ***P < 0.001). Given the strong correlation between factors within the various variables, the physical indicates, available nutrients, total nutrients and microbial activity, diversity, and community factors were downscaled by a principal component analysis before PLS-PM and the correlation analysis. A bar chart visualizes total effects based on PLS-PM **(b)**.

The structural equation model examining the interrelationships among soil environmental factors, bacterial diversity, bacterial community composition, and SQI revealed that soil nutrients (SOM, TN, NO_3_^-^-N, NH_4_^+^-N, and Olsen-P), microbial activity (MBN, MBC), bacterial community composition, and diversity all had direct positive effects on SQI (**Figure 8a**). Bacterial diversity was negatively influenced by pH (SPC = -0.518*). Meanwhile, total soil nutrients indirectly positively affected SQI by influencing microbial activity (SPC = 0.562***) and bacterial community composition (SPC = 0.784***). These pathways indicate that the increase in bacterial diversity was driven by changes in pH, NO_3_^-^-N, Olsen-P, and NH_4_^+^-N. Finally, the total effects of each soil environmental factor, as well as microbial community and diversity, on SQI are presented (**Figure 8b**).

## 4 Discussion

### 4.1 The addition of manure and straw improves soil quality by increasing soil nutrient levels

Studies have shown that, compared with conventional fertilization, the addition of manure and straw significantly improves soil physicochemical properties (Tian et al., 2022). The increases in TN, Olsen-P, NH_4_^+^-N, NO_3_^-^-N, SOC, MBN, and MBC under the MNP and SNP treatments are consistent with previous findings on the application of organic materials (**Table 1**). For instance, a 30-year long-term experiment on black soil demonstrated that organic manure partial substitution for chemical fertilizer and straw partial substitution significantly increased soil organic carbon by 58.7% and 10.7%, total nitrogen by 66.4% and 18.5%, respectively, compared with chemical fertilizer alone (Zhang et al., 2025). Notably, the MNP treatment exhibited significantly higher levels of Olsen-P, NO_3_^-^-N, SOC, and MBN than the SNP treatment, indicating that manure has a greater advantage over straw in providing stable organic matter and available nutrients. The observed decreases in soil pH and bulk density following manure and straw addition create a more favorable habitat for microbial activity (Guo et al., 2010). Furthermore, the SQI and maize yield under the MNP and SNP treatments were significantly higher than those under NP, with the MNP treatment showing better performance. Meanwhile, MBC, SOC, TN, and Olsen-P were identified as the primary contributors to SQI (**Figure 2a**), while SOC, TN, MBC, and NO_3_^-^-N were the primary contributors to maize yield (**Figure 2b**). These findings confirm the important roles of SOC, TN, and MBC in soil quality and crop production. This conclusion has also been confirmed in studies examining green manure management practices (Lyu et al., 2025a). In conclusion, the addition of manure and straw effectively enhances soil quality and crop yield by improving soil physicochemical properties, increasing microbial biomass, and optimizing soil nutrient status. Among these, the MNP treatment outperformed the SNP treatment, primarily due to the advantages of manure in increasing available nutrients (especially Olsen-P and NO_3_^-^-N) and microbial biomass.

**Table 1:** Soil properties under different fertilization practices.

Year	Treatments	pH	BD (g·cm^-3^)	TN (g·kg^-1^)	Olsen-P (mg·kg^-1^)	NH_4_^+^-N (mg·kg^-1^)	NO_3_^--^N (mg·kg^-1^)	SOC (g·kg^-1^)	MBN (mg·kg^-1^)	MBC (mg·kg^-1^)	C/N
2024	CK	8.59 ± 0.01a	1.27 ± 0.03a	0.83 ± 0.04f	2.72 ± 0.33e	1.1 ± 0.07d	4.3 ± 0.29f	8.16 ± 0.25e	31.93 ± 1.94e	288.77 ± 19.19d	9.85 ± 0.52ab
	M	8.36 ± 0.05b	1.19 ± 0.02cd	1.23 ± 0.05c	10.38 ± 0.46d	2.35 ± 0.14b	10.18 ± 0.57c	10.79 ± 0.55c	44.68 ± 0.96b	345.59 ± 6.34b	8.76 ± 0.4b
	MNP	8.23 ± 0.02c	1.17 ± 0.04d	1.71 ± 0.07a	39.61 ± 1.18a	3.06 ± 0.05a	20.8 ± 0.44a	16.24 ± 0.34a	54.52 ± 1.36a	383.01 ± 8.39a	9.53 ± 0.56ab
	N	8.57 ± 0.02a	1.25 ± 0.03ab	0.93 ± 0.04e	2.5 ± 0.21e	1.23 ± 0.05c	6.51 ± 0.3e	8.46 ± 0.28e	37.62 ± 0.85d	310.38 ± 8.24c	9.1 ± 0.48ab
	NP	8.36 ± 0.03b	1.22 ± 0.02bc	1.05 ± 0.04d	14.27 ± 1.01c	1.26 ± 0.05c	8.98 ± 0.62d	9.61 ± 0.34d	41.86 ± 1.44c	335.19 ± 9.27b	9.18 ± 0.53ab
	SNP	8.4 ± 0.02b	1.1 ± 0.03e	1.4 ± 0.07b	25.14 ± 0.63b	2.93 ± 0.11a	12.25 ± 0.65b	14.11 ± 1.1b	47.11 ± 1.6b	375.92 ± 11.7a	10.13 ± 1.04a
2025	CK	8.48 ± 0.03a	1.31 ± 0.03a	0.88 ± 0.03d	1.67 ± 0.16e	1.17 ± 0.17d	4.74 ± 0.46e	7.52 ± 0.25e	32.81 ± 2.19c	231.21 ± 15.46f	8.57 ± 0.26b
	M	8.28 ± 0.04c	1.2 ± 0.02c	1.13 ± 0.02b	9.01 ± 1.11d	2.24 ± 0.25b	10.85 ± 0.84bc	10.64 ± 0.51c	46.48 ± 0.71b	325.25 ± 10.68c	9.42 ± 0.37ab
	MNP	8.29 ± 0.05c	1.18 ± 0.02c	1.54 ± 0.05a	40.78 ± 4.8a	3.12 ± 0.08a	20 ± 0.72a	15.94 ± 0.44a	55.63 ± 3.15a	397.63 ± 12.67a	10.38 ± 0.51a
	N	8.41 ± 0.02ab	1.26 ± 0.03b	0.97 ± 0.08cd	2.01 ± 0.28e	1.47 ± 0.32c	6.72 ± 0.13d	8.54 ± 0.39d	43.56 ± 0.93b	275.42 ± 10.86e	8.83 ± 1.05b
	NP	8.39 ± 0.05b	1.27 ± 0.01ab	1.03 ± 0.08bc	13.8 ± 1.3c	1.28 ± 0.12c	9.64 ± 0.58c	9.79 ± 0.35c	44.01 ± 0.67b	304.32 ± 9.86d	9.55 ± 1.02ab
	SNP	8.28 ± 0.06c	1.12 ± 0.03d	1.51 ± 0.05a	25.29 ± 1.47b	2.87 ± 0.13a	11.59 ± 1.08b	14.94 ± 0.82b	45.42 ± 1.07b	349.76 ± 9.25b	9.91 ± 0.6ab

pH, potential of hydrogen; BD, soil bulk density; TN, total nitrogen; Olsen-P, available phosphorus; NH_4_^+^-N, ammonium nitrogen; NO_3_^--^N, nitrate nitrogen; SOC, soil organic carbon; MBC, soil microbial biomass carbon; MBN, soil microbial biomass nitrogen; C/N, SOC/TN. Different letters indicate significant difference at *P* < 0.05 for treatments.

### 4.2 The addition of manure and straw increased the abundance of dominant bacteria and bacterial community diversity

In this study, *Pseudomonadota*, *Acidobacteriota*, *Planctomycetota*, *Bacteroidota*, and *Actinomycetota* were identified as the dominant bacterial phyla in the soil bacterial community (**Figure 3a**), which is consistent with previous findings on soil microbial community composition under organic material application (Kong et al., 2011; Yang et al., 2026). Compared with the NP treatment, the relative abundances of *Pseudomonadota* under the MNP and SNP treatments increased by 12.36% and 15.44%, respectively. *Pseudomonadota* are a dominant group widely found in organic-rich soils, and their metabolic diversity enables them to secrete various extracellular enzymes, thereby effectively decomposing complex organic compounds in the soil (Breitkreuz et al., 2020; Thonar et al., 2017). The synergistic interaction between manure/straw addition and microorganisms including *Pseudomonadota* promotes the mineralization of organic nitrogen and organic phosphorus, thereby enhancing soil nutrient availability (Pereira and Castro, 2014). Notably, the relative abundance of *Acidobacteriota* under the CK treatment was higher than that under the MNP and SNP treatments by 23.22% and 20.23%, respectively. *Acidobacteriota* are generally considered oligotrophic bacteria that are more competitive in nutrient-poor soils (Kielak et al., 2016). This finding further confirms that organic material addition alters the ecological strategy of bacterial communities from oligotrophic to copiotrophic groups by increasing soil nutrient levels (Wang et al., 2020). At the genus level, *Sphingomonas* and *Lysobacter* were dominant in the MNP and SNP treatments (**Supplementary Figure 1a**), both of which have been reported to possess the ability to degrade complex organic compounds and suppress soil-borne pathogens, indicating that organic material addition enriches beneficial functional microbial taxa (Gomes et al., 2009; Pinyakong et al., 2003). In summary, the addition of manure and straw regulates the composition of soil bacterial communities, enriching functional microbial taxa associated with nutrient transformation, organic matter decomposition, and disease suppression, while inhibiting oligotrophic microorganisms such as Acidobacteriota. These changes in community structure collectively contribute to the improvement of soil quality and crop productivity.

Similar to previous studies, the Mantel test results revealed that environmental factors significantly influenced bacterial community composition but not fungal community composition (**Figure 6**), indicating that bacterial communities are more sensitive to changes in the soil environment (Kong et al., 2011). Research has shown that bacteria are far more efficient than fungi in utilizing simple organic compounds, whereas fungi are more efficient in utilizing complex compounds, which may be an important reason for the differential responses of these two microbial groups to environmental factors (Fang et al., 2024). Specifically, from an ecological perspective, fungi possess extensive hyphal networks that enable long-distance translocation of water, nutrients, and carbon, thereby buffering against the effects of local-scale environmental heterogeneity (Simonin et al., 2012). Furthermore, the slower turnover rates and longer generation times of fungi compared with bacteria confer greater intrinsic stability to fungal communities under short- to medium-term fertilization regimes. Meanwhile, fungi exhibit high functional redundancy, particularly among saprotrophic taxa involved in decomposition processes, which may buffer the community against compositional shifts even when the activity of specific functional groups is suppressed (Banerjee et al., 2016). From a methodological standpoint, primer selection and sequencing depth may also influence the detectability of fungal community responses. Although the ITS2 region is widely used and offers good resolution for Ascomycota and Basidiomycota, it may introduce amplification biases against certain fungal lineages and may fail to detect rare or low-abundance taxa that are more sensitive to environmental changes (Bellemain et al., 2010). Notably, with the exception of pH and bulk density, all environmental factors exhibited significant or extremely significant positive correlations with one another, indicating that the addition of manure and straw comprehensively improves the soil environment by reducing bulk density and modulating pH, thereby promoting the enhancement of soil nutrients (Khan et al., 2020).

Compared with CK and NP, the MNP treatment significantly increased soil bacterial diversity (**Figure 4a**), which is consistent with previous findings that manure application increases bacterial alpha diversity. For instance, a 14-year long-term field experiment demonstrated that manure substitution for chemical fertilizers significantly enhanced soil bacterial α-diversity compared with urea alone, with manure treatments showing superior soil quality index and yield stability. Similarly, a long-term greenhouse experiment reported that vermicompost application significantly increased the Shannon index of soil bacteria compared with chemical fertilizer alone (Chen et al., 2022; Qu et al., 2025; Zhao et al., 2026b). Regression analysis indicated that the increase in soil available nutrients (NO_3_^-^-N, Olsen-P, and NH_4_^+^-N) and the decrease in soil pH were the main reasons for the increase in bacterial diversity. Notably, the MNP and SNP treatments significantly reduced soil fungal diversity. The increase in bacterial diversity can be attributed to the introduction of substantial labile carbon substrates through organic material addition, which activated dormant copiotrophic bacterial taxa (such as *Pseudomonadota* and *Bacteroidetes*) in the soil. Meanwhile, the nitrogen and phosphorus supplied by manure application alleviated stoichiometric constraints, broadened the ecological niche breadth, and facilitated the coexistence of multiple bacterial species. In contrast, the decline in fungal diversity may be explained by the manure-induced shift of the soil environment toward a high-nutrient, low-lignin state, which suppressed specialist lignocellulose-degrading fungi (e.g., *Basidiomycota* and *Ascomycota*). Concurrently, the introduction of exogenous microorganisms via manure intensified interspecific competition, thereby eroding the resident fungal diversity. The reduction in fungal diversity may compromise hyphal entanglement capacity and impair macro-aggregate stability, whereas the enhanced bacterial diversity accelerated the mineralization of labile organic matter and nutrient turnover, functionally compensating for the immediate nutrient supply to crops (Yang et al., 2026). Regression analysis showed that soil bacterial diversity was positively correlated with Olsen-P, SOC, TN, MBN, NO_3_^-^-N, NH_4_^+^-N, and MBC, while negatively correlated with soil pH (**Figure 7**). This finding further confirms the conclusion that organic material addition promotes bacterial diversity by optimizing the soil chemical environment (Liu et al., 2024). In summary, the addition of manure and straw exerts differential effects on bacterial and fungal diversity. This differential response may contribute to the formation of a more stable and efficient microbial ecosystem, in which the diversification of bacterial communities enhances functional redundancy in nutrient cycling, while the streamlining of fungal communities may reduce substrate competition, thereby collectively improving soil quality and crop productivity.

### 4.3 The addition of organic fertilizer and crop straw increased both the abundance of beneficial microbial communities and bacterial diversity, thereby improving soil quality and increasing crop yields

The important role of microbial community structure and diversity in maintaining nutrient cycling has been widely recognized (Zheng et al., 2019). In this study, a structural equation model was constructed, and the results indicated that soil nutrients, microbial activity, bacterial community composition, and diversity all had direct positive effects on the soil quality index. This finding is consistent with previous results from this study, further confirming the central roles of soil nutrients, microbial activity, and bacterial community characteristics in regulating soil quality (Lyu et al., 2025a). Notably, bacterial diversity was negatively influenced by pH, indicating that the addition of manure and straw increases bacterial diversity by reducing soil pH. Higher microbial diversity implies greater functional redundancy and resistance to disturbance, enabling more stable maintenance of nutrient cycling and organic matter decomposition processes. This finding is consistent with previous studies showing that exogenous organic matter input reduces pH by increasing soil organic matter and microbial biomass, thereby alleviating alkalization stress and creating a favorable living environment for a wider range of microbial taxa (Gao et al., 2021). Furthermore, total soil nutrients indirectly and positively affected the soil quality index by influencing microbial activity and bacterial community composition. This suggests that total nutrients not only directly reflect soil fertility but also indirectly drive soil quality improvement by regulating the function and structure of microbial communities (Borase et al., 2020). In summary, the addition of manure and straw directly or indirectly influences bacterial community composition and diversity by regulating soil physicochemical properties, thereby enhancing soil quality. Among these, bacterial community composition contributes more to the soil quality index than diversity, indicating that the enrichment of dominant functional microbial taxa plays a more critical role in regulating soil health.

### 4.4 Outlook

In a long-term field experiment, the total dataset method was employed to evaluate the responses of different organic material management strategies to soil quality and crop yield, and to identify the main factors influencing soil quality. Furthermore, this study elucidated the microbiological mechanisms by which manure and straw addition affect soil quality from the perspective of changes in microbial community composition and diversity. These results provide important parameters for the rational application of organic materials (manure and straw) in sustainable agriculture and for soil quality assessment. In the future, we plan to continue employing techniques such as metagenomics, stable isotope probing, and high-throughput sequencing to comprehensively elucidate the species composition, functional potential, metabolic pathways of soil microbial communities, as well as their interactions with environmental factors and crops, thereby providing a deeper theoretical basis for the optimization of organic fertilization practices and the regulation of soil health.

## 5 Conclusion

This study indicates that compared with NP, both MNP and SNP significantly improved the SQI and maize yield, with the MNP treatment showing better effects than the SNP treatment. The improvement in soil quality under the MNP treatment is primarily attributed to the enhancement and optimization of soil physicochemical properties. Among these, SOC, TN, and MBC were identified as the main contributors to SQI and yield. Regarding microbial community characteristics, the MNP and SNP treatments increased the abundance of dominant bacterial communities (such as *Sphingomonas* and *Lysobacter* under *Pseudomonadota*) by raising soil total nutrient levels. Additionally, these treatments increased bacterial diversity by regulating soil pH and available nutrient contents. In conclusion, the addition of manure and straw effectively enhances soil quality and crop yield by optimizing soil physicochemical properties, increasing microbial biomass, and regulating bacterial community composition and diversity, with the combined application of manure and chemical fertilizers achieving the best comprehensive effects.

## Data Availability

The data presented in the study are deposited in the NCBI repository, accession number PRJNA1465624.
